# Sustainable fall prevention across Europe: challenges and opportunities

**DOI:** 10.1007/s40520-022-02178-w

**Published:** 2022-07-13

**Authors:** Nathalie van der Velde, Lotta Seppala, Mirko Petrovic, Jesper Ryg, Maw Pin Tan, Manuel Montero-Odasso, Finbarr C. Martin, Tahir Masud

**Affiliations:** 1 Amsterdam UMC, Department of Internal Medicine, Section of Geriatric Medicine, University of Amsterdam, Amsterdam, The Netherlands; 2 Amsterdam Public Health Research Institute, Amsterdam, The Netherlands; 3 Section of Geriatrics, Department of Internal Medicine and Paediatrics, Ghent University, Ghent, Belgium; 4 Department of Geriatric Medicine, Odense University Hospital, Odense, Denmark; 5 Geriatric Research Unit, Department of Clinical Research, University of Southern Denmark, Odense, Denmark; 6 Division of Geriatric Medicine, Department of Medicine, University of Malaya, Kuala Lumpur, Malaysia; 7 Department of Medical Sciences, School of Healthcare and Medical Sciences, Sunway University, Subang Jaya, Malaysia; 8 Gait and Brain Lab, Parkwood Institute, Lawson Health Research Institute, London, ON Canada; 9 Faculty of Life Sciences and Medicine, Population Health Sciences, King’s College London, London, UK; 10 Nottingham University Hospitals NHS Trust, Nottingham, UK

**Keywords:** Fall prevention, Injury prevention, Geriatric, Older persons, Pragmatic trials, European

Falls and related injuries form a growing health-care problem in aging societies. Between 40 and 60% of older fallers in the last year report being injured [1]. Around 15–20% of falls result in serious (non-fracture) injuries including fractures [2]. Non-injurious falls have also been associated with adverse health effects, including accelerated functional decline, anxiety and depression, fear of falling, and social withdrawal [3]. Consequently, fall incidents have an impact on societal health-care expenditure, equaling 0.85–1.5% of the total health-care expenditure in Western countries [4].

To tackle this health-care issue, many countries with developed health-care services have established fall prevention services. Given its multifactorial nature, it is assumed that comprehensive geriatric assessment (CGA) leading to individually targeted interventions would be effective. Previous literature has shown that several good quality trials have resulted in a reduction in falls [5]. Despite local differences, these services generally address (1) risk stratification and (2) multifactorial assessment (MA) of risk factors and (3) accompanying interventions. Accordingly, several medical societies and organizations have published clinical practice guidelines for fall prevention and management and a recent systematic review found a high degree of agreement in several areas [6]. These guideline components are consistent with a recent systematic review and network meta-analyses showing that several single and multiple fall prevention interventions are associated with reduction in falls [7].

However, in the real world, total deaths and disability-adjusted life years (DALYs) due to falls have increased steadily since 1990 [3], largely explained by demographic change accompanied by increased prevalence of multimorbidity, polypharmacy, and frailty [8]. When standardized for age, different patterns in incident and DALY rate change from 1990 to 2017 have been observed between countries and regions [8, 9]. One of the explaining factors could be the challenge of translating research findings into clinical practice at scale. The research-to-implementation gap is not only applicable to falls, but also to many areas where intervention involves individual behavior, such as exercise recommendations. As Tinetti et al. had already stated in 2006, challenges such as time constraints and competing clinical demands are similar to those affecting other health-care services, although perhaps of greater magnitude for fall prevention because of its personnel-intensive nature. Furthermore, there is a general lack of knowledge, skills and coordination necessary for the unique nature of geriatric syndromes, which do not fit the traditional disease model of clinical care and reimbursement [10]. Reducing the incidence of falls is further hindered by the lack of robust population-level policies [11]. The WHO Step Safely report advocates a systems approach with the interplay of complex factors: persons’ biology, behavior, physical environment, and the cultural and socioeconomic environment within a bio-psycho-social model. It recommends interventions encompassing safer personal situation, safer environments, safer policies, and better legislation. Effective policies require engagement with appropriate stakeholders, which should include decision- and policymakers, health-care funders, health-care professionals, and older peoples’ associations and advocates [11].

Besides the financial and knowledge gaps that might endanger sustainable fall prevention policies, there are also the negative findings of two recent large, pragmatic trials [12, 13] that might impact investment and implementation of fall services. However, adding these negative trials to the recent systematic review and network meta-analyses on fall preventive interventions did not alter the initial results, i.e., the evidence base for fall prevention remains strong [3]. Nevertheless, the taskforce experts of the World Falls Guidelines initiative [3] are concerned that these findings might be misinterpreted by stakeholders as proof of ineffectiveness rather than proof of inefficacy of the trial interventions. A careful interpretation of all the current evidence including these recent trials is of major importance. These trials illustrated that within current health-care systems, it is difficult to successfully implement interventions proven to be effective in previous smaller research trial settings [3]. Therefore, we provide a summary on relevant items of both the trials that need to be acknowledged when translating the findings to clinical practice.

The pragmatic PreFIT trial [12] was a negative trial in which neither fractures nor falls were reduced by the two intervention arms (exercise and multifactorial risk assessment) compared to the mail-only advice arm. However, we should be cautious in generalizing the conclusions of the trial because of different study-related aspects explained below. The results do not mean that exercise or multifactorial assessment (MA) followed by appropriate interventions cannot work. It means that the intervention arms delivered in this trial did not reduce fall and fracture risks or rates. There are several potential reasons for this, including inclusion rates, chosen target population, comparative risk, participation rate, intervention design, uptake and adherence and fidelity.

Approximately, 34% of 70+ patients of participating general practitioner (GP) practices in the UK agreed to participate. This was followed by risk screening and within the intervention arms those at high risk were offered the interventions. For risk screening, an earlier developed questionnaire was used, which addresses history of falls, difficulty of balancing whilst walking or dressing and difficulty with activities of daily living [12]. However, an important part of the AGS/BGS algorithm [14] is objective gait assessment, and it is unclear how this relates to assessing balance problems through a postal survey. Therefore, the screening procedure may have identified participants at average lower risk of falls compared to the original AGS/BGS guideline method. The participation rate in both arms was relatively low. In the exercise arm (1): 36.9% (*n* = 1079) were eligible for treatment, but of those 32.4% (*n* = 350) did not attend the sessions. Similarly, in the multifactorial intervention arm (2), 37.5% (1074) were eligible for treatment, but of those 40.9% did not attend the sessions. As to uptake of those attending, there are also concerns. For example, within the multifactorial arm, an important intervention concerns polypharmacy and particularly fall risk increasing drugs. It is therefore noteworthy that the rates of prescribing of psychotropic or related medications was 18.1% at baseline, rising to 18.8% over the study period with no difference observed between the groups, suggesting that at least this aspect of medication-related risk was not successfully addressed.

Furthermore, as to fidelity, in those study participants who attended the sessions, the exercise was of insufficient intensity, progression, adherence, and duration as compared to the original Otago program. The mean number of exercise sessions attended (5.5, SD 1.98) was less than the minimum of seven (face to face and telephone) recommended. There was evidence of probable efficacy of the exercise, however, as most [391 of 454 (86%)] improved or remained at the ceiling upper level of the Otago Exercise Program strength scale and 330 of 453 (72%) of improved or maintained the top level of balance. However, if a 25% reduction in falls was achieved in this final group, then the relative drop for the 3310 enrolled would already be fairly small.

Regarding the second intervention arm, the ideal MA and intervention program should use a multidisciplinary CGA approach, which requires input from trained—in fall assessment—physicians, nurses, physiotherapists, and occupational therapists. Insufficient details were offered on the personnel who performed the assessments and to what extent all the MAs that should have been administered were actually completed. Further, details on the delivery of interventions following the MAs were not available. The above, therefore, limits the level of confidence in the fidelity of MA and subsequent interventions in this trial compared to previous trials that have shown that this approach can effectively reduce falls.

The pragmatic US STRIDE trial [13] was also a negative trial since the risk of a serious adjudicated fall injury was not significantly lower in the intervention arm, although the secondary outcome rate of self-reported fall-related injuries was. The intervention was a standardized seven-item fall risk assessment leading to an individual care plan of protocolized interventions delivered through existing primary care services, with no additional resources provided. As for PreFIT, it is important that we should be cautious in generalizing the conclusions of STRIDE. The findings do not mean that MA, followed by appropriate interventions do not work. It means that the interventions delivered in this trial did not reduce risk of serious, adjudicated fall injuries. Again, the potential reasons are inclusion rates, the target population, comparative risk, participation rate, intervention design, uptake and adherence and fidelity.

Approximately, 29% of patients aged 70+ years from participating practices were deemed eligible and provided informed consent. The participation rate was relatively low, 14% of those randomized to the intervention arm not receiving it. The intervention was administered by nurses trained with an online course with just one face-to-face session and motivational interviewing training. The nurse assessment contrasts with earlier trials which took multidisciplinary approaches both to assessment and interventions, including physiotherapists, physicians, occupational therapists, and/or pharmacists skilled in their own professional competencies. In comparison, the Winchester falls project showed that a structured multidisciplinary assessment of recurrent fallers significantly reduced the number of participants experiencing further falls, but a community-based nurse-led assessment with targeted referral to other professionals did not [15].

Participants were supported to prioritise up to three identified risk factors and then accept the relevant intervention. It appears that the potential for interventions was both under-recognized by the assessments and under-prioritized by participants. For example, usage of fall-risk increasing drugs (FRIDs) was identified in only 34% of participants, although earlier studies have shown that the prevalence of FRIDs use in older community dwellers is up to 93%, and earlier trials were able to de-prescribe one or more FRIDs in over 50% of participants [16]. Moreover, only 29% of those taking FRIDs agreed to have this addressed. Similarly, only half the participants who had a home safety hazard agreed to actions designed to mitigate this risk.

Other potential barriers such as transportation, co-payments, or insurance cover may also have contributed to low uptake and adherence. Adherence to behavioral modification was not monitored. Although nearly all participants accepted interventions for at least one risk factor, participants, study nurses or their physicians may have chosen easier applicable, but less effective interventions such as vitamin D supplementation.

Furthermore, when generalizing the findings from these RCTs, it is good to remember that the participants included in the studies may not represent the real-world patients. Frailer patients may be less willing to participate in such trials and for example the majority of the participants in these trials had no cognitive impairment [12, 13].

In summary, the PreFIT and the STRIDE trials [12, 13] suggest that both exercise and MA strategies that have been shown to reduce falls in previous trials cannot easily be applied with sufficient fidelity through current existing services in the UK and the USA, respectively. There is marked heterogeneity across countries and regions in the provision of falls services. The results of these studies should therefore not be used to justify decommissioning of well-established fall services where the interventions are delivered in line with previous successful evidence-based fall prevention studies. On the contrary, these trial outcomes stress the importance of the provision of sufficient resources to roll out and support high-quality sustainable deliverance of fall prevention programs that are in line with previous good-quality successful trials. Further research is needed to assess how best to implement such developments most cost-effectively. It is likely that the enhanced services would cost more to the prevention service provider, but less to the health and social care system if sufficient falls, fractures, and other injuries are prevented thereby reducing hospital admissions and ongoing need for social care.

Successful implementation of health-care intervention such as fall prevention depends on many factors at different health-care levels including the (1) innovation, (2) individual professional, (3) patient, (4) social context, (5) organizational context, and (6) the economic and political context [17]. For successful and durable implementation of fall services, collaboration between relevant medical disciplines, health-care insurers and governmental bodies is essential. To facilitate this, the Special Interest Group on Falls and Fracture prevention of the European Geriatric Medicine Society is currently preparing an international survey on current practices in fall prevention services throughout Europe to determine gaps and opportunities, identify best practices, and relevant stakeholders for sustainable fall prevention for older persons. Also, to achieve global consensus on the optimal content of fall preventive interventions and to facilitate knowledge distribution, the above-mentioned task force of worldwide experts was installed in 2019 at the first World Falls and Postural Stability Conference in Kuala Lumpur, Malaysia, to produce the first World Falls Prevention and Management Guidelines [3]. The guidelines will include overall recommendations and more specific ones with regard to assessment, risk stratification, and interventions in different settings and risk groups. This global consensus is expected to be an important incentive to implement the newest available evidence and expert knowledge into practice globally and these guidelines are expected to be published later this year (2022). The guidelines will be useful in facilitating negotiations with policymakers and financers to guide further development and consolidation of falls services throughout the world.
